# Higher glucose, insulin and insulin resistance (HOMA-IR) in childhood predict adverse cardiovascular risk in early adulthood: the Pune Children’s Study

**DOI:** 10.1007/s00125-015-3602-z

**Published:** 2015-05-05

**Authors:** Chittaranjan S. Yajnik, Prachi A. Katre, Suyog M. Joshi, Kalyanaraman Kumaran, Dattatray S. Bhat, Himangi G. Lubree, Nilam Memane, Arun S. Kinare, Anand N. Pandit, Sheila A. Bhave, Ashish Bavdekar, Caroline H. D. Fall

**Affiliations:** Kamalnayan Bajaj Diabetology Research Centre, Diabetes Unit, King Edward Memorial Hospital Research Centre, Rasta Peth, Pune, 411011 India; MRC Lifecourse Epidemiology Unit, University of Southampton, Southampton, UK; Department of Pediatrics, King Edward Memorial Hospital Research Centre, Pune, India

**Keywords:** Cardiovascular risk, Childhood insulin resistance, Diabetes, Indians, Young adults

## Abstract

**Aims/hypothesis:**

The Pune Children’s Study aimed to test whether glucose and insulin measurements in childhood predict cardiovascular risk factors in young adulthood.

**Methods:**

We followed up 357 participants (75% follow-up) at 21 years of age who had undergone detailed measurements at 8 years of age (glucose, insulin, HOMA-IR and other indices). Oral glucose tolerance, anthropometry, plasma lipids, BP, carotid intima–media thickness (IMT) and arterial pulse wave velocity (PWV) were measured at 21 years.

**Results:**

Higher fasting glucose, insulin and HOMA-IR at 8 years predicted higher glucose, insulin, HOMA-IR, BP, lipids and IMT at 21 years. A 1 SD change in 8 year variables was associated with a 0.10–0.27 SD change at 21 years independently of obesity/adiposity at 8 years of age. A greater rise in glucose–insulin variables between 8 and 21 years was associated with higher cardiovascular risk factors, including PWV. Participants whose HOMA-IR measurement remained in the highest quartile (*n* = 31) had a more adverse cardiovascular risk profile compared with those whose HOMA-IR measurement remained in the lowest quartile (*n* = 28).

**Conclusions/interpretation:**

Prepubertal glucose–insulin metabolism is associated with adult cardiovascular risk and markers of atherosclerosis. Our results support interventions to improve glucose–insulin metabolism in childhood to reduce cardiovascular risk in later life.

**Electronic supplementary material:**

The online version of this article (doi:10.1007/s00125-015-3602-z) contains peer-reviewed but unedited supplementary material, which is available to authorised users.

## Introduction

Since the demonstration of an association between birth size and risk of type 2 diabetes and cardiovascular disease (CVD) by Hales et al [1], a number of birth cohorts have been established to document the life course evolution of chronic non-communicable diseases [2–5]. Some of these cohorts showed an inverse association between birthweight and childhood risk factors for type 2 diabetes and CVD [6, 7]. Few of these prospective birth cohorts have reported follow-ups extending into adult life.

It is recognised that a small elevation in plasma glucose concentration in adults (lower than that defining diabetes) is associated with an increased risk of later CVD [8–10]. This has led to the creation of a ‘borderline’ group of ‘prediabetes’ and the concept of ‘dysglycaemia’ [11–13]. It is generally believed that the increased CVD risk in such a situation is related to ‘insulin resistance’, although some believe that ‘hyperinsulinaemia’ itself is responsible [14–18]. A number of studies have demonstrated tracking of CVD risk factors from childhood through to adulthood [19–22]. However, there are very few studies which can relate childhood glucose and insulin measurements to future risk of CVD. The Bogalusa Heart Study showed childhood glucose and insulin concentrations to be predictive of adult diabetes and CVD risk factors [23, 24]. However, the ‘childhood’ measurements spanned across puberty and extended into early adulthood (4–18 years in one study and 5–23 years in the other), follow-up rates were low, and the ‘adult’ measurements were spread over a wide age range (19–39 years). Demonstration of an association between prepubertal insulin resistance and adult CVD risk will allow us to highlight the importance of this phase in the life course evolution of CVD risk.

India has high rates of diabetes [25] and in comparison with Western populations Indians develop diabetes at a younger age and lower BMI. A national survey in India demonstrated a prevalence of 2.4% diabetes and 11.5% impaired glucose tolerance in young people aged 20–29 years, and rates of gestational diabetes in young women are high at ∼15% [26, 27]. Indians as a group are one of the most insulin-resistant populations in the world [28] and it is thought that this contributes to their high risk of diabetes and CVD. There is little life course data to support this assumption.

The Pune Children’s Study was established in 1991 to examine the associations between birthweight and risk factors for diabetes and CVD in later life. At 4 and 8 years of age, we found an inverse association between birthweight and glucose, insulin and HOMA-IR [2, 6]. We have followed up these children at 21 years of age and now examine the possibility that insulin resistance in childhood will be associated with future CVD risk.

## Methods

**Fig. 1:**
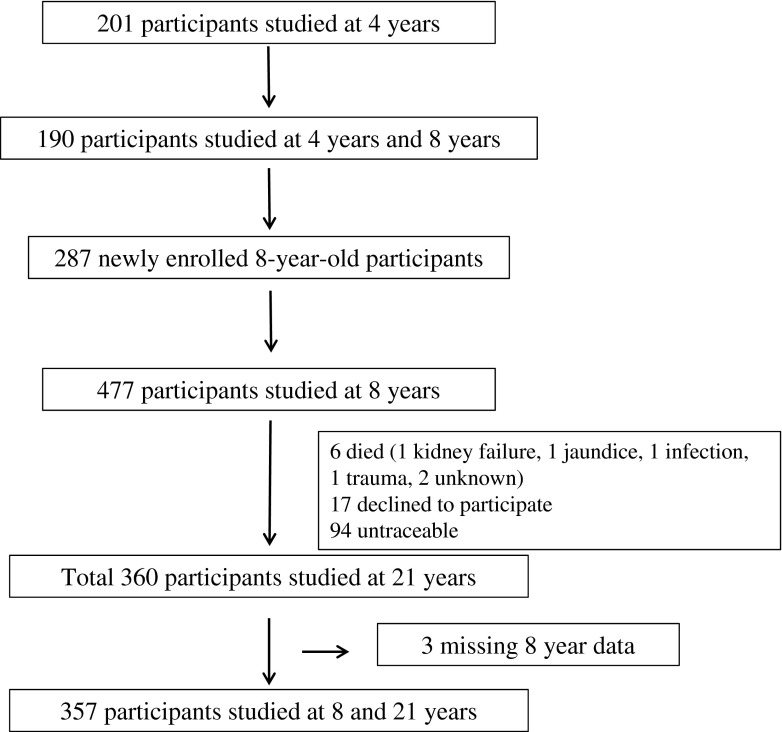
Flow diagram showing enrolment and follow-up in the Pune Children’s Study

The Pune Children’s Study has been described previously [2, 6]. Children born in the King Edward Memorial Hospital, Pune, India, between 1987 and 1989 were studied during childhood and again at the age of 21 years (Fig. 1). Ethics permission for the study was obtained from the King Edward Memorial Hospital Ethics Committee and informed written consent was obtained from all participants.

The procedures used at 8 and 21 years were similar. The participants were admitted to the Diabetes Unit, King Edward Memorial Hospital, the evening before the investigations and fasted overnight after a standard dinner. Anthropometry was measured according to standardised protocols [6]. All children were examined at 8 years by a paediatrician for pubertal assessment. Acanthosis nigricans was examined in the neck and axillary regions by a trained observer and graded [29]. Grades 0 and 1 were rated as absence of acanthosis and grades ≥2 were rated as presence of acanthosis; the grade was used as a dichotomous variable in the statistical analysis. BP was measured in the supine position after 15 min of rest, using an oscillometric method (Dinamap; Critikon, Tampa, FL, USA [at 8 years]; UA-767PC; A & D Instruments, Abingdon, UK [at 21 years]). The average of two readings 5 min apart was used. At 21 years, intima–media thickness (IMT, a surrogate for atherosclerosis) was measured by one observer (ASK) using ProSound Alpha 7 (Aloka, Tokyo, Japan) (linear array probe 5–13 MHz) at the bifurcation of the common carotid arteries [30]. IMT measurements were carried out on longitudinal sections in the common carotid segment 1 cm proximal to the bifurcation. A section showing maximum IMT on the posterior wall was considered for documentation and the average of three readings was taken. The observer also examined for the presence of plaques. Intra-observer CV was <3%. We measured pulse wave velocity (PWV) in the brachial arteries using a non-invasive Windows-based oscillometric technique (PeriScope; Genesis Medical Systems, Chennai, India). This instrument has been validated for PWV measurements [31]. PWV is a measure of arterial compliance and is increased in stiffer arteries. Intra- and inter-observer CVs were <2% and <10%, respectively. The average of the measurements from right and left was used for analysis of IMT and PWV.

The next morning, fasting venous blood samples were taken for measurement of plasma glucose, insulin and lipids. An OGTT was carried out according to the WHO protocol, using a dose of 1.75 g anhydrous glucose per kg body weight at 8 years and 75 g at 21 years. Blood samples were collected for measurement of glucose and insulin at 30 and 120 min.

### Laboratory analyses

Plasma glucose, cholesterol, HDL-cholesterol and triacylglycerol concentrations were measured using standard enzymatic methods (Spectrum; Abbott, Irving, TX, USA [at 8 years]; Hitachi 902; Roche Diagnostics, Mannheim, Germany [at 21 years]). Between-batch CVs for all these assays were <3% in the normal range. Plasma insulin, proinsulin and 32–33 split proinsulin were measured using a Delfia technique (Victor 2; Wallac, Turku, Finland) at 8 years and a two-site immunoenzymometric assay (Medgenix, Fleurus, Belgium) at 21 years; between-batch CVs for insulin measurements were <6%. We recalculated HOMA-IR and HOMA-β at both 8 years and 21 years by the current accepted standard (online Oxford HOMA calculator: available from www.dtu.ox.ac.uk, accessed 15 July 2012) for consistency. There was an excellent correlation (*r* = 0.99, *p* < 0.001) with HOMA-IR calculated by the formula [32]. Insulin secretion was measured as the insulinogenic index (increment in plasma insulin divided by that in plasma glucose at 30 min) [33]. The Matsuda index of insulin sensitivity was computed by the formula:

$$ k/\surd \left(\mathrm{fasting}\kern0.5em \mathrm{glucose}\times 120\  \min \kern0.5em \mathrm{glucose}\times \mathrm{fasting}\kern0.5em \mathrm{insulin}\times 120\  \min \kern0.5em \mathrm{insulin}\right), $$

where *k* (constant) = 10,000 [34].

### Definitions

BMI was used to define overweight (≥25 and <30 kg/m^2^) and obesity (≥30 kg/m^2^) [35]. Glycaemic classification was done according to ADA (75 g OGTT) criteria: impaired fasting glucose (IFG) as fasting plasma glucose 5.6–6.9 mmol/l and 120 min plasma glucose <7.8 mmol/l; impaired glucose tolerance (IGT) as fasting plasma glucose <5.6 mmol/l and 120 min plasma glucose 7.8–11.0 mmol/l; and diabetes mellitus as fasting plasma glucose ≥7.0 mmol/l or 120 min plasma glucose ≥11.1 mmol/l [11]. We defined prediabetes as IFG and/or IGT. We defined hypercholesterolaemia (≥5.1 mmol/l) according to National Cholesterol Education Program criteria [36], and hypertriglyceridaemia (≥1.7 mmol/l), low HDL-cholesterol (<1.03 mmol/l for male participants and <1.29 mmol/l for female participants) and hypertension (≥130/85 mmHg) by International Diabetes Federation criteria [12].

### Statistical methods

Variables with skewed distributions (insulin and triacylglycerol concentrations, HOMA-IR, Matsuda index, HOMA-β, insulinogenic index and IMT) were log-transformed to achieve normality for analysis. The primary analysis was to study associations of childhood glucose and insulin measurements with CVD risk factors at 21 years of age. We tested for linear and non-linear associations using regression. We performed model checks by carrying out residual analysis and confirmed the normality of residuals [37]. Both the exposure and outcome variables were standardised to age- and sex-specific *z* scores. The regression coefficients (*β*) therefore represent the SD change in outcome per SD change in exposure. Glucose, insulin, BMI and skinfold thickness at 8 years were strongly interrelated and could therefore confound associations with 21 year CVD risk factors. Therefore, we also used residuals in these associations to test their independent relationships. For example, the residual of 8 year fasting glucose, regressed upon 8 year fasting insulin, BMI and skinfold thickness, was tested in addition to fasting glucose and so on. We adjusted for multiple testing using the standard Bonferroni correction (0.05 divided by number of outcomes [17] = 0.003) and used this value for statistical significance. We also calculated the sensitivity, specificity, predictive values and receiver operating characteristic (ROC) AUC to assess the relevance for clinical practice of high HOMA-IR in childhood predicting CVD risk in young adulthood.

We performed sensitivity analyses between participants and non-participants using regression imputation. We developed an imputation model using the variables significantly associated with each other at 8 years in a multiple regression model. We applied this regression imputation for participants to calculate 21 year data and compared the imputed values with available values. We imputed 21 year BMI using 8 year age, BMI, total cholesterol, triacylglycerol, fasting insulin and HOMA-IR as predictors. As there were no significant differences between observed and imputed values for participants (indicating ‘missing at random’), we used this imputation model to calculate 21 year data for the non-participants. We then compared the observed values of the participants with the imputed values of the non-participants.

Analysis was carried out using SPSS software (version 16.0; SPSS, Chicago, IL, USA).

## Results

### General characteristics

The characteristics of the 357 participants (191 boys) at 8 and 21 years of age are shown in Table 1. Glucose and insulin concentrations and HOMA-IR at 8 years were no different in these participants compared with those in the 120 who did not participate in the 21 year follow-up. Non-participants had higher 8 year BMI (14.0 vs 13.6 kg/m^2^, *p* = 0.05) and greater skinfold thickness (*p* < 0.05, all) compared with participants. Participants’ 21 year BMI showed no differences with imputed values for non-participants (21.6 vs 21.9 kg/m^2^; *p* > 0.05).

**Table 1:** Characteristics of the participants at 8 and 21 years of age

Characteristic	8 years		21 years	
	Male	Female	Male	Female
*N*	191	166	191	166
Age, years	8.4 (0.1)	8.4 (0.1)	21.4 (0.4)	21.4 (0.4)
Height, cm	124.7 (10.6)	124.3 (6.1)	172.0 (6.6)	156.9 (6.4)***
Weight, kg	21.6 (3.6)	21.1 (3.9)	65.3 (13.1)	52.3 (10.6)***
BMI, kg/m^2^	13.7 (1.5)	13.5 (1.7)	22.0 (4.0)	21.2 (4.1)
WHR	0.82 (0.04)	0.82 (0.05)	0.86 (0.05)	0.79 (0.05)***
Fasting glucose, mmol/l	4.6 (0.6)	4.5 (0.6)	5.1 (0.6)	5.0 (0.4)*
30 min glucose, mmol/l	7.2 (1.4)	7.5 (1.4)	8.3 (1.3)	7.7 (1.4)***
120 min glucose, mmol/l	5.1 (4.2, 5.6)	5.1 (4.3, 5.7)	5.5 (4.8, 6.5)	5.7 (4.8, 6.5)
Fasting insulin^a^, pmol/l	23.0 (16.0, 35.0)	26.0 (19.0, 35.0)	41.4 (26.1, 64.2)	48.0 (31.8, 68.4)
30 min insulin^a^, pmol/l	173.5 (92.7, 268.2)	183.0 (119.7, 275.0)	495.6 (362.5, 705.6)	505.0 (327.4, 727.9)
120 min insulin^a^, pmol/l	76.0 (40.0, 118.0)	98.0 (54.0, 167.0)**	297.0 (172.6, 488.2)	302.0 (199.5, 491.4)
HOMA-IR^a^	0.5 (0.3, 0.7)	0.6 (0.4, 0.7)	0.9 (0.6, 1.4)	1.0 (0.7, 1.5)
Matsuda index^a^	17.0 (11.7, 26.4)	13.9 (9.3, 20.4)*	6.0 (3.8, 8.9)	5.2 (3.5, 8.0)
HOMA-β^a^	75.2 (56.4, 94.8)	76.1 (62.2, 98.3)	85.1 (60.2, 106.1)	94.5 (72.4, 122.5)**
Insulinogenic index^a^	19.3 (11.0, 32.5)	20.6 (13.5, 31.4)	52.2 (37.8, 78.2)	58.1 (39.1, 83.1)
Systolic BP, mmHg	109.8 (14.1)	107.9 (19.5)	115.3 (10.1)	102.9 (9.1)***
Diastolic BP, mmHg	62.6 (9.7)	61.5 (13.8)	65.4 (8.4)	63.6 (7.9)*
Cholesterol, mmol/l	3.4 (0.6)	3.4 (0.6)	3.8 (0.7)	3.7 (0.7)
Triacylglycerols^a^, mmol/l	0.7 (0.5, 0.9)	0.7 (0.6, 0.9)	0.8 (0.6, 1.2)	0.7 (0.6, 1.0)***
HDL-cholesterol, mmol/l	1.1 (0.2)	1.0 (0.3)	0.9 (0.1)	1.1 (0.2)***
IMT^a^, mm	Not measured	Not measured	0.37 (0.34, 0.42)	0.34 (0.32, 0.37)***
PWV^a^, cm/s	Not measured	Not measured	1,010.8 (947.3, 1,095.5)	885.0 (827.3, 958.6)***

Values are mean (SD) or ^a^median (25th–75th centiles) for skewed variables

*p* values were tested using ANOVA or the Mann–Whitney *U* test as appropriate

*p < 0.05, **p < 0.01, ***p < 0.001

At 21 years, 66 participants (18.5%) were overweight (21% of boys and 15% of girls) and nine (2.5%) were obese (four boys and five girls). Fifteen (4.2%) were hypertensive, 21 (5.8%) had high cholesterol, 27 (7.5%) had high triacylglycerols, and 248 (69%) had low HDL-cholesterol. Three were already known to have diabetes (all on insulin treatment); we diagnosed five new cases of diabetes and 61 of prediabetes (40 IFG and 21 IGT) at this follow-up. Acanthosis nigricans was diagnosed in 42 (11.7%) at the age of 21 years. All but six had a Framingham Risk Score of ≤3.

### Sex differences

There were no differences in characteristics between boys and girls at 8 years for anthropometric and biochemical variables. All were considered prepubertal by the paediatrician on clinical assessment. At 21 years, men were taller, heavier and had higher glycaemia and CVD risk factors compared with women (Table 1). We present sex-adjusted results, as there was no effect modification by sex.

### Associations between childhood exposures and adulthood outcomes

#### Cardiometabolic risk factors (Tables 2 and 3)

**Table 2:** Associations of 8 year and 21 year glucose and insulin variables

8 year variable	21 year variable									
	BMI	WHR	Fasting glucose	120 min glucose	Fasting insulin	120 min insulin	HOMA-IR	Matsuda index	Insulinogenic index	HOMA-β
Fasting glucose	0.06 (−0.03, 0.17)	−0.03 (−0.13, 0.07)	0.14 (0.04, 0.25)	0.10 (0.00, 0.21)	0.12 (0.02, 0.23)	0.10 (0.00, 0.21)	0.13 (0.03, 0.24)	−0.15^†^ (−0.26, −0.05)	0.02 (−0.07, 0.13)	0.06 (−0.03, 0.17)
Residual of fasting glucose on fasting insulin, BMI and skinfold thickness	0.04 (−0.08, 0.17)	−0.01 (−0.14, 0.11)	0.15^†^ (0.06, 0.24)	0.07 (−0.05, 0.20)	0.11 (−0.01, 0.24)	0.04 (−0.08, 0.17)	0.13 (0.01, 0.26)	−0.11 (−0.24, −0.01)	0.01 (−0.11, 0.14)	0.04 (−0.08, 0.17)
Fasting insulin	0.27^†^ (0.17, 0.38)	0.18^†^ (0.08, 0.28)	0.16^†^ (0.06, 0.27)	0.08 (−0.02, 0.19)	0.14 (0.03, 0.24)	0.15 (0.04, 0.25)	0.14 (0.04, 0.25)	−0.17^†^ (−0.28, −0.07)	0.07 (−0.03, 0.18)	0.07 (−0.03, 0.17)
Residual of fasting insulin on fasting glucose, BMI and skinfold thickness	0.07 (−0.02, 0.18)	0.18^†^ (0.08, 0.29)	0.07 (−0.00, 0.15)	0.09 (−0.00, 0.20)	0.11 (0.01, 0.22)	0.16 (0.06, 0.27)	0.12 (0.01, 0.22)	−0.17^†^ (−0.27, −0.06)	0.04 (−0.05, 0.15)	0.08 (−0.01, 0.19)
HOMA-IR	0.26^†^ (0.16, 0.36)	0.15^†^ (0.04, 0.25)	0.16^†^ (0.06, 0.27)	0.09 (−0.03, 0.20)	0.14 (0.03, 0.24)	0.15 (0.05, 0.26)	0.14 (0.04, 0.25)	−0.18^†^ (−0.28, −0.07)	0.08 (−0.02, 0.18)	0.07 (−0.03, 0.17)
Residual of HOMA-IR on BMI and skinfold thickness	0.08 (−0.02, 0.19)	0.08 (−0.02, 0.19)	0.09 (−0.01, 0.19)	0.11 (0.00, 0.21)	0.12 (0.02, 0.23)	0.16^†^ (0.05, 0.27)	0.13 (0.02, 0.23)	−0.18^†^ (−0.29, −0.08)	0.04 (−0.06, 0.14)	0.09 (−0.01, 0.19)
HOMA-β	0.22^†^ (0.11, 0.32)	0.19^†^ (0.08, 0.30)	0.05 (−0.05, 0.16)	0.01 (−0.10, 0.12)	0.04 (−0.06, 0.15)	0.06 (−0.04, 0.17)	0.04 (−0.07, 0.15)	−0.05 (−0.17, 0.05)	0.04 (−0.07, 0.15)	0.02 (−0.08, 0.13)
Matsuda index	−0.20^†^ (−0.31, −0.09)	0.08 (−0.02, 0.19)	−0.01 (−0.12, 0.09)	−0.13 (−0.24, −0.02)	−0.08 (−0.19, 0.02)	−0.15 (−0.26, −0.05)	−0.08 (−0.19, 0.02)	0.17^†^ (0.07, 0.28)	−0.04 (−0.15, 0.06)	−0.07 (−0.18, 0.03)
Insulinogenic index	0.20^†^ (0.09, 0.30)	−0.08 (−0.18, 0.02)	−0.06 (−0.14, 0.01)	0.07 (−0.03, 0.18)	0.04 (−0.06, 0.15)	0.18^†^ (0.08, 0.29)	0.04 (−0.06, 0.14)	−0.13 (−0.24, −0.02)	0.06 (−0.04, 0.16)	0.08 (−0.01, 0.19)

Values are *β* regression coefficients (95% CI). All dependent and independent variables in a linear regression model are *z*-standardised

^†^ *p* < 0.003

**Table 3:** Associations of 8 year glucose and insulin variables and 21 year CVD risk factors

8 year variable	21 year variable						
	Systolic BP	Diastolic BP	Cholesterol	Triacylglycerols	HDL-cholesterol	IMT	PWV
Fasting glucose	−0.18^†^ (−0.28, −0.08)	−0.02 (−0.13, 0.07)	0.15 (0.04, 0.25)	0.07 (−0.03, 0.17)	0.06 (−0.03, 0.17)	0.13 (0.01, 0.26)	−0.09 (−0.23. 0.03)
Residual of fasting glucose on fasting insulin, BMI and skinfold thickness	−0.11 (−0.24, 0.01)	−0.06 (−0.19, 0.06)	0.09 (−0.03, 0.22)	0.07 (−0.05, 0.20)	0.06 (−0.06, 0.19)	0.14 (0.01, 0.27)	−0.10 (−0.24, 0.03)
Fasting insulin	0.12 (0.02, 0.23)	0.14 (0.04, 0.25)	0.05 (−0.04, 0.16)	0.16^†^ (0.05, 0.26)	−0.15 (−0.26, −0.05)	0.06 (−0.04, 0.16)	−0.03 (−0.14, 0.07)
Residual of fasting insulin on fasting glucose, BMI and skinfold thickness	0.15 (0.05, 0.26)	0.08 (−0.02, 0.09)	−0.04 (−0.14, 0.06)	0.12 (0.01, 0.23)	−0.17^†^ (−0.27, −0.06)	0.03 (−0.07, 0.13)	0.02 (−0.08, 0.13)
HOMA-IR	0.08 (−0.02, 0.18)	0.13 (0.02, 0.24)	0.07 (−0.03, 0.17)	0.14 (0.03, 0.24)	−0.13 (−0.24, −0.03)	0.03 (−0.07, 0.13)	0.003 (−0.10, 0.11)
Residual of HOMA-IR on BMI and skinfold thickness	0.03 (−0.07, 0.14)	0.05 (−0.05, 0.16)	0.02 (−0.08, 0.12)	0.11 (0.00, 0.22)	−0.13 (−0.23, −0.02)	0.04 (−0.06, 0.15)	0.007 (−0.10, 0.12)
HOMA-β	0.23^†^ (0.12, 0.34)	0.15 (0.04, 0.26)	−0.04 (−0.14, 0.06)	0.09 (−0.01, 0.20)	−0.18^†^ (−0.29, −0.07)	−0.04 (−0.15, 0.06)	0.05 (−0.06, 0.17)
Matsuda index	0.22^†^ (0.11, 0.33)	−0.02 (−0.13, 0.08)	−0.19^†^ (−0.30, −0.08)	−0.05 (−0.16, 0.05)	−0.03 (−0.14, 0.07)	0.10 (−0.00, 0.21)	−0.04 (−0.16, 0.06)
Insulinogenic index	−0.13 (−0.23,−0.02)	0.06 (−0.04, 0.16)	0.09 (−0.00, 0.20)	0.05 (−0.05, 0.16)	−0.03 (−0.13, 0.07)	−0.04 (−0.15, 0.06)	0.04 (−0.06, 0.16)

Values are *β* regression coefficients (95% CI). All dependent and independent variables in a linear regression model are *z*-standardised

^†^ *p* < 0.003

Fasting glucose at 8 years was directly related to 21 year fasting and 120 min glucose and insulin concentrations, HOMA-IR and cholesterol, and inversely to Matsuda index and systolic BP; a 1 SD change was associated with a 0.10–0.18 SD change. Eight year fasting glucose had no significant associations with BMI and WHR at 21 years. The residual of 8 year glucose (independently of insulin, BMI and skinfold thickness at 8 years) was significantly associated with fasting glucose, HOMA-IR and IMT at 21 years.

Fasting insulin at 8 years was directly related to 21 year BMI, WHR, fasting glucose, fasting insulin, 120 min insulin, HOMA-IR, BP and triacylglycerols, and inversely to Matsuda index and HDL-cholesterol; a 1 SD change was associated with a 0.12–0.27 SD change. The residual of 8 year insulin (independently of glucose, BMI and skinfold thickness) showed similar associations with 21 year insulin, systolic BP and lipid variables. Insulin concentrations were directly related to proinsulin and 32–33 split proinsulin concentrations (*r* = ∼0.4, *p* < 0.001). Both proinsulin and split proinsulin concentrations at 8 years were associated with a range of CVD risk factors very similar to the associations of insulin, but not independent of insulin concentrations (data not shown).

Associations of HOMA-IR at 8 years were similar to those of 8 year fasting insulin (Tables 2 and 3). Eight year HOMA-IR was not related to either the presence or grade of acanthosis nigricans at 21 years (*p* = 0.254). Individuals in the highest quartile of HOMA-IR at 8 years had a higher risk of being in the highest quartile of the following CVD risk factors at 21 years: BMI, WHR, 120 min insulin, HOMA-IR, systolic and diastolic BP and triacylglycerols (RR 1.48–1.81; *p* < 0.05, all) (Table 4) compared with those in the lowest quartile. Similarly, they were also more likely to be hyperglycaemic (IFG + IGT + diabetes) at 21 years (RR 1.86; 95% CI 0.96, 3.60). Sensitivity and positive predictive values were low (<40%), but specificity and negative predictive values were in the range of 70–80%. ROC AUC values ranged from 50% to 65%.

**Table 4:** RR, sensitivity, specificity, predictive values and ROC AUC of being in the upper quartile of CVD risk factors at 21 years if in the upper quartile of HOMA-IR at 8 years

Variable	RR (95% CI)	Sensitivity (%)	Specificity (%)	Positive predictive value (%)	Negative predictive value (%)	ROC AUC (%)
BMI	1.81 (1.26, 2.60)	38.3	78.8	37.9	79.1	63.4
WHR	1.73 (1.20, 2.48)	37.2	78.4	36.7	78.7	62.3
Fasting glucose	1.35 (0.92, 1.99)	30.6	76.1	31.0	75.8	58.7
120 min glucose	1.17 (0.78, 1.76)	28.9	75.9	27.5	76.4	54.1
Fasting insulin	1.41 (0.96, 2.07)	32.9	76.6	32.1	77.2	60.5
120 min insulin	1.54 (1.06, 2.22)	34.4	77.6	34.8	77.3	60.0
HOMA-IR	1.65 (1.14, 2.38)	36.4	77.7	35.6	78.4	60.0
Matsuda index	1.46 (1.00, 2.13)	33.7	77.0	33.7	77.0	62.0
Insulinogenic index	0.95 (0.61, 1.45)	25.0	73.7	24.1	74.6	52.0
HOMA-β	0.54 (0.31, 0.92)	15.8	70.9	14.9	72.4	56.0
Systolic BP	1.48 (1.01, 2.15)	33.2	77.1	33.3	77.4	59.0
Diastolic BP	1.50 (1.03, 2.19)	34.1	77.2	33.3	77.8	56.7
Cholesterol	1.25 (0.84, 1.87)	30.1	75.8	28.7	77.0	53.2
Triacylglycerols	1.55 (1.07, 2.25)	34.8	77.5	34.8	77.8	61.1
HDL-cholesterol	1.28 (0.87, 1.88)	30.6	76.1	31.0	75.8	55.9
IMT	1.14 (0.73, 1.79)	29.8	73.8	27.4	76.1	55.0
PWV	1.19 (0.77, 1.84)	28.7	75.8	27.2	77.1	52.0

Associations of HOMA-β, Matsuda index and insulinogenic index at 8 years with 21 years are shown in Tables 2 and 3.

Fasting glucose, fasting insulin and HOMA-IR at 21 years were significantly associated with a range of concurrent CVD risk factors (electronic supplementary material [ESM] Table 1). Fasting insulin and HOMA-IR at 21 years were associated with acanthosis nigricans (*p* = 0.001, both), and those with acanthosis nigricans had higher BMI, WHR and other CVD risk factors (ESM Table 2). The 21 year associations were stronger than the corresponding associations of the 8 year measurements (Tables 2 and 3).

We compared 21 year CVD risk factors in those who were in the highest quartile of HOMA-IR at both 8 and 21 years with those who remained in the lowest quartile at both time points. Those who remained in the highest quartile (*n* = 31) had a more adverse CVD risk profile compared with those who remained in the lowest quartile (*n* = 28) (ESM Table 3).

Four year measurements were available for 147 participants. Four year HOMA-IR was not significantly associated with CVD risk factors at 21 years of age (data not shown), although the direction of association and the effect size (*β*) were similar to those at 8 years of age.

#### Vascular markers of atherosclerosis (Table 3)

We did not observe any significant plaques in any participant. Fasting glucose at 8 years and its residual were significantly associated with IMT at 21 years. There were no relationships between 8 year glucose–insulin variables and PWV at 21 years. Higher glucose–insulin at 21 years and a higher change in these variables between 8 and 21 years were associated with higher PWV but not IMT. A 1 SD change in glucose, insulin and HOMA-IR was associated with a 0.08–0.13 SD change in PWV (*p* < 0.05, all).

### Adjustments for dietary intake, physical activity and socioeconomic status

In addition to age, sex, BMI and WHR, we adjusted our models for diet (total calorie and macronutrient intakes), physical activity and socioeconomic status at 21 years. On adjusting for physical activity, the relationships between 8 year glucose, insulin and HOMA-IR and 21 year insulin and HOMA-IR became non-significant. Adjusting for dietary intake and socioeconomic class made no difference.

There was no evidence of non-linear associations in our analyses.

## Discussion

The Pune Children’s Study provides the first report of associations in India between glucose and insulin variables in childhood and a range of adult CVD risk factors including vascular markers of atherosclerosis. Expectedly, at the young age of 21 years, there were only a few participants with diabetes, hypertension and dyslipidaemia, and none had had a cardiovascular event. Sixty-nine (19.4%) had elevated glucose concentrations (eight diabetic, 61 prediabetic). Fasting glucose, insulin and HOMA-IR at 8 years of age were related to glycaemic variables and CVD risk factors at 21 years of age independently of 8 year adiposity. Glucose–insulin at 21 years and the change in glucose–insulin between 8 and 21 years had a stronger association with 21 year CVD outcomes. Glucose at 8 years (but not insulin and HOMA-IR) was associated with carotid IMT, while 21 year glucose–insulin and change between 8 and 21 years were associated with PWV.

A number of cross-sectional studies in children and adults, as well as prospective studies in adults, have reported associations of glucose, insulin and insulin resistance with CVD risk factors [38–40]. The cross-sectional nature of these studies, short-term follow-up and inability to account for pre-existing pathology make it difficult to determine causality. There are only a few prospective studies of CVD risk factors from childhood to adulthood and few intervention trials in childhood to test prevention across the life course. The best known is the Bogalusa Heart Study, which studied children into adulthood with serial measurements of CVD risk factors to study associations and predictions. Among 1,606 children who were measured in 1981–1982 and 1988–1991, those with insulin levels in the highest quartile in both surveys had higher BMI, triacylglycerols, LDL- and VLDL-cholesterol, systolic and diastolic BP, and lower HDL-cholesterol at the second survey compared with those with insulin in the lowest quartile in both surveys. They also had higher incident obesity, hypertension and dyslipidaemia [24]. In a 17 year follow-up of the Bogalusa Heart Study, those with incident prediabetes and diabetes were more likely to have been in the top decile for glucose, insulin and HOMA-IR during childhood [23]. Our findings for prediabetes and diabetes are similar and provide stronger evidence to consider active intervention in childhood to prevent later diabetes and CVD. It is of note that children in the Bogalusa Heart Study cohort were enrolled across a wide age range, spanning different pubertal stages, and the follow-up rate was low (∼10–15% of the cohort were followed up at all time points). Moreover, the age range at follow-up was broad (19–39 years) and ‘disease’ outcomes were ascertained in only ∼5% of the original cohort. In another prospective study, remarkable for its serial euglycaemic clamp measurements in children at 13, 15 and 19 years of age, insulin resistance and fasting insulin concentrations at age 13 were predictive of triacylglycerol concentrations, BP and a metabolic syndrome score at 19 years, independently of BMI [41]. This suggests that insulin resistance is an independent risk factor for diabetes and CVD.

Broadly, the strength of associations of 8 year glucose, insulin and HOMA-IR with 21 year CVD risk factors was similar, although insulin was associated with a wider range of CVD risk factors compared with glucose. Given the observational design and close interrelationships of these factors, we are unable to comment on the relative importance of glucose, insulin and insulin resistance for future outcomes. Opinion is also divided as to whether glucose or insulin concentrations, or insulin resistance, are at the root of these associations. Glucose might operate through its downstream metabolic pathways (‘glucotoxicity’) either in the pancreatic beta cells or in the other tissues of the body. There is an interesting possibility that requirement of insulin in certain tissues for the entry of glucose might modulate these effects such that different tissues are differently affected [42]. Reaven suggested that insulin resistance is the root cause of type 2 diabetes and CVD [15], based on cross-sectional observational studies in adults. Insulin resistance implies a derangement in various intracellular pathways and these may affect metabolism, cellular growth and proliferation in various tissues (endothelium, renal nephrons, sympathetic nervous system and liver), contributing to increased CVD risk [43]. Recent research has highlighted a possible role for epigenetic mechanisms in the pathogenesis of vascular complications of diabetes [44]. In our study, we found an interesting difference in the associations of markers of atherosclerosis. IMT, a structural marker, was associated only with 8 year glucose, while PWV, a functional marker, was not associated with any 8 year measurements but with 21 year measurements and with change between 8 and 21 years in both glucose and insulin variables. Hyperglycaemia has been associated with increased PWV, which may be partly due to the accumulation of AGEs in elastin resulting in fracture of elastin and consequently stiffer arteries [45]. Lack of association with IMT may be due to the small variability at this young age of our participants. The exact mechanisms involved need further investigation. In adults there is evidence of a beneficial effect of lifestyle intervention on the risk of diabetes [46, 47], although not always on CVD. In our study, the confounding effect of physical activity suggests a possible role for lifestyle interventions.

Our results suggest that the effect of childhood glucose–insulin metabolism on adult CVD risk operates across the ‘normal’ range in a continuous manner. Those who tracked as the most insulin resistant from childhood to adulthood had a significantly worse CVD risk profile compared with those who tracked as the most insulin sensitive, similar to the observations in the Bogalusa Heart Study. This is an important observation to support a life course model for CVD risk and it extends its generalisability from affluent populations to non-obese, developing populations. The lack of association between 4 year and 21 year measurements may be because of inadequate power. Although sensitivity, specificity and predictive values were generally low, a 1 SD increase in HOMA-IR resulted in an increase in outcomes ranging from 0.1 mmol/l for glucose to 1.1 kg/m^2^ for BMI. Our study provides biologically important information and further work is necessary to translate these findings into clinical practice for identifying high risk children.

Our study has many strengths. Serial measurements allowed us to examine the impact of childhood exposures on adult outcomes. A narrow age range (CV <2%) and prepubertal and postpubertal status at the two time points allowed us to study CVD risk independently of these potential confounders. In a meta-analysis, early puberty was associated with higher adult BMI and risk of CVD [48]. Our results reinforce the life course evolution of CVD risk before the onset of puberty. Our loss to follow-up of ∼25% is one of the lowest for a long-term longitudinal study and adds to its internal validity. Skinfold thickness measurements allowed us to adjust for adiposity (body fat) instead of only BMI. The procedures for measurements at 8 and 21 years were comparable and were supervised by the same observer (HGL). Sensitivity analyses using regression imputation confirmed a lack of differential bias due to follow-up loss.

There are a few limitations. The participants were born in one hospital in Pune, which may limit generalisability. However, the hospital is the second largest in Pune and offers obstetric services to people from a wide range of socioeconomic classes, thus improving representation. Rates of prediabetes in our cohort were comparable to data from other urban Indian studies [26–28]. Being an observational study, causality cannot be established. Lack of multiple measurements of exposure compromises assertion of temporality. However, as we were examining the predictivity of 8 year glycaemic variables on 21 year outcomes, we believe there is a strong suggestion of temporal relationships. Despite measurement of a number of confounders, residual confounding cannot be ruled out. Our interpretation of insulin resistance is predominantly based on the HOMA model and not on euglycaemic clamp studies, which would be impossible in our situation. However, Matsuda index (a dynamic measure) [34] gave similar findings.

In summary, this is the first report of life course associations of CVD risk in young non-obese adults from a developing country. Higher glucose, insulin and insulin resistance in childhood and a higher change in glucose–insulin between childhood and young adulthood were associated with higher CVD risk factors in early adulthood. Those who were persistently insulin resistant had a higher CVD risk. Our previous demonstration of an association between childhood insulin resistance and low birthweight suggests that insulin resistance may be on the pathway between fetal growth and CVD risk. These findings reinforce the need for interventions in early life to improve glucose–insulin metabolism to curtail the rapidly escalating epidemics of diabetes and CVD. This may be achieved by promoting healthy eating habits and physical activity. Controlling excess weight gain in childhood may be beneficial [49, 50]. Policy-makers should invest in the health of children to improve the future health of the nation. This is important because the prevention strategy is currently focused on secondary and tertiary prevention. The Nobel laureate economist James J. Heckman demonstrated that investment early in life has substantially higher returns than later interventions [51].

## Electronic supplementary material

ESM Table 1 (PDF 75 kb)

ESM Table 2 (PDF 84.4 kb)

ESM Table 3 (PDF 85 kb)

## Abbreviations

CVD: Cardiovascular disease
IFG: Impaired fasting glucose
IGT: Impaired glucose tolerance
IMT: Intima–media thickness
PWV: Pulse wave velocity
ROC: Receiver operating characteristic

## References

[1] Hales CN, Barker DJ, Clark PM (1991). Fetal and infant growth and impaired glucose tolerance at age 64. BMJ.

[2] Yajnik CS, Fall CH, Vaidya U (1995). Fetal growth and glucose and insulin metabolism in four year-old Indian children. Diabet Med.

[3] Rao S, Yajnik CS, Kanade A (2001). Intake of micronutrient-rich foods in rural Indian mothers is associated with the size of their babies at birth: Pune Maternal Nutrition Study. J Nutr.

[4] Inskip HM, Godfrey KM, Robinson SM (2006). Cohort profile: the Southampton Women's Survey. Int J Epidemiol.

[5] Richter LM, Victora CG, Hallal PC (2012). Cohort profile: the consortium of health-orientated research in transitioning societies. Int J Epidemiol.

[6] Bavdekar A, Yajnik CS, Fall CH (1999). Insulin resistance syndrome in 8 year-old Indian children: small at birth, big at 8 years, or both?. Diabetes.

[7] Krishnaveni GV, Veena SR, Wills AK, Hill JC, Katat SC, Fall CH (2010). Adiposity, insulin resistance and cardiovascular risk factors in 9-10 year-old Indian children: relationships with birth size and postnatal growth. J Dev Orig Health Dis.

[8] Welborn TA, Wearne K (1979). Coronary heart disease incidence and cardiovascular mortality in Busselton with reference to glucose and insulin concentrations. Diabetes Care.

[9] Ducimetiere P, Eschwege E, Papoz L, Richard JL, Claude JR, Rosselin G (1980). Relationship of plasma insulin levels to the incidence of myocardial infarction and coronary heart disease mortality in a middle-aged population. Diabetologia.

[10] Pyorala K, Savolainen E, Kaukola S, Haapakoski J (1985). Plasma insulin as coronary heart disease risk factor: relationship to other risk factors and predictive value during 9½ year follow-up of the Helsinki Policemen Study population. Acta Med Scand Suppl.

[11] American Diabetes Association (2014). Standards of medical care in diabetes—2014. Diabetes Care.

[12] Alberti KG, Zimmet P, Shaw J (2006). Metabolic syndrome—a new world-wide definition. A consensus statement from the International Diabetes Federation. Diabet Med.

[13] Gerstein HC, Bosch J, Dagenais GR (2012). Basal insulin and cardiovascular and other outcomes in dysglycaemia. N Engl J Med.

[14] Stout RW (1990). Insulin and atheroma. 20-yr perspective. Diabetes Care.

[15] Reaven GM (1988). Banting lecture 1988. Role of insulin resistance in human disease. Diabetes.

[16] Corkey BE (2012). Banting lecture 2011: hyperinsulinemia: cause or consequence?. Diabetes.

[17] Jiang X, Srinivasan SR, Bao W, Berenson GS (1993). Association of fasting insulin with blood pressure in young individuals. The Bogalusa Heart Study. Arch Intern Med.

[18] Manolio TA, Savage PJ, Burke GL (1990). Association of fasting insulin with blood pressure and lipids in young adults. The CARDIA study. Arteriosclerosis.

[19] Juhola J, Magnussen CG, Viikari JS (2011). Tracking of serum lipid levels, blood pressure, and body mass index from childhood to adulthood: the Cardiovascular Risk in Young Finns Study. J Pediatr.

[20] Twisk JW, Kemper HC, van Mechelen W, Post GB (1997). Tracking of risk factors for coronary heart disease over a 14 year period: a comparison between lifestyle and biologic risk factors with data from the Amsterdam Growth and Health Study. Am J Epidemiol.

[21] Akerblom HK, Viikari J, Raitakari OT, Uhari M (1999). Cardiovascular Risk in Young Finns Study: general outline and recent developments. Ann Med.

[22] Joshi SM, Katre PA, Kumaran K (2014). Tracking of cardiovascular risk factors from childhood to young adulthood—the Pune Children's Study. Int J Cardiol.

[23] Nguyen QM, Srinivasan SR, Xu JH, Chen W, Kieltyka L, Berenson GS (2010). Utility of childhood glucose homeostasis variables in predicting adult diabetes and related cardiometabolic risk factors: the Bogalusa Heart Study. Diabetes Care.

[24] Bao W, Srinivasan SR, Berenson GS (1996). Persistent elevation of plasma insulin levels is associated with increased cardiovascular risk in children and young adults. The Bogalusa Heart Study. Circulation.

[25] Guariguata L, Whiting DR, Hambleton I, Beagley J, Linnenkamp U, Shaw JE (2014). Global estimates of diabetes prevalence for 2013 and projections for 2035. Diabetes Res Clin Pract.

[26] Ramachandran A, Snehalatha C, Kapur A (2001). High prevalence of diabetes and impaired glucose tolerance in India: National Urban Diabetes Survey. Diabetologia.

[27] Seshiah V, Diabetes in Pregnancy Study Group India (2010). Fifth National Conference of Diabetes in Pregnancy Study Group, India. J Assoc Physicians India.

[28] McKeigue PM, Shah B, Marmot MG (1991). Relation of central obesity and insulin resistance with high diabetes prevalence and cardiovascular risk in South Asians. Lancet.

[29] Burke JP, Hale DE, Hazuda HP, Stern MP (1999). A quantitative scale of acanthosis nigricans. Diabetes Care.

[30] Baldassarre D, Amato M, Bondioli A, Sirtori CR, Tremoli E (2000). Carotid artery intima–media thickness measured by ultrasonography in normal clinical practice correlates well with atherosclerosis risk factors. Stroke.

[31] Naidu MU, Reddy BM, Yashmaina S, Patnaik AN, Rani PU (2005). Validity and reproducibility of arterial pulse wave velocity measurement using new device with oscillometric technique: a pilot study. Biomed Eng Online.

[32] Matthews DR, Hosker JP, Rudenski AS, Naylor BA, Treacher DF, Turner RC (1985). Homeostasis model assessment: insulin resistance and beta-cell function from fasting plasma glucose and insulin concentrations in man. Diabetologia.

[33] Wareham NJ, Phillips DI, Byrne CD, Hales CN (1995). The 30 minute insulin incremental response in an oral glucose tolerance test as a measure of insulin secretion. Diabet Med.

[34] DeFronzo RA, Matsuda M (2010). Reduced time points to calculate the composite index. Diabetes Care.

[35] World Health Organization. BMI classification. Global database on body mass index. Available from www.who.int/bmi. Accessed 15 Jul 2012

[36] National Cholesterol Education Program Expert Panel on Detection, Evaluation and Treatment of High Blood Cholesterol in Adults (Adult Treatment Panel III), 2002. (Publication no. NIH 02:5215). National Institutes of Health, Bethesda, MD

[37] Willett WC, Howe GR, Kushi LH (1997). Adjustment for total energy intake in epidemiological studies. Am J Clin Nutr.

[38] Modan M, Halkin H, Almog S (1985). Hyperinsulinemia. A link between hypertension obesity and glucose intolerance. J Clin Invest.

[39] Li C, Ford ES, Zhao G, Mokdad AH (2009). Prevalence of pre-diabetes and its association with clustering of cardiometabolic risk factors and hyperinsulinemia among U.S. adolescents: National Health and Nutrition Examination Survey 2005-2006. Diabetes Care.

[40] Goran MI, Lane C, Toledo-Corral C, Weigensberg MJ (2008). Persistence of pre-diabetes in overweight and obese Hispanic children: association with progressive insulin resistance, poor beta-cell function, and increasing visceral fat. Diabetes.

[41] Sinaiko AR, Steinberger J, Moran A, Hong CP, Prineas RJ, Jacobs DR (2006). Influence of insulin resistance and body mass index at age 13 on systolic blood pressure, triglycerides, and high-density lipoprotein cholesterol at age 19. Hypertension.

[42] Brownlee M (2005). The pathobiology of diabetic complications: a unifying mechanism. Diabetes.

[43] DeFronzo RA, Ferrannini E (1991). Insulin resistance. A multifaceted syndrome responsible for NIDDM, obesity, hypertension, dyslipidemia, and atherosclerotic cardiovascular disease. Diabetes Care.

[44] Reddy MA, Zhang E, Natarajan R (2015). Epigenetic mechanisms in diabetic complications and metabolic memory. Diabetologia.

[45] Goldin A, Beckman JA, Schmidt AM, Creager MA (2006). Advanced glycation end products. Sparking the development of diabetic vascular injury. Circulation.

[46] Dunkley AJ, Bodicoat DH, Greaves CJ (2014). Diabetes prevention in the real world: effectiveness of pragmatic lifestyle interventions for the prevention of type 2 diabetes and of the impact of adherence to guideline recommendations: a systematic review and meta-analysis. Diabetes Care.

[47] Gerstein HC, Yusuf S, Bosch J (2006). Effect of rosiglitazone on the frequency of diabetes in patients with impaired glucose tolerance or impaired fasting glucose: a randomised controlled trial. Lancet.

[48] Prentice P, Viner RM (2013). Pubertal timing and adult obesity and cardiometabolic risk in women and men: a systematic review and meta-analysis. Int J Obes.

[49] Taylor RW, McAuley KA, Barbezat W, Farmer VL, Williams SM, Mann JI (2008). Two year follow-up of an obesity prevention initiative in children: the APPLE project. Am J Clin Nutr.

[50] Waters E, de Silva-Sanigorski A, Hall BJ, et al (2011) Interventions for preventing obesity in children. Cochrane Database Syst Rev, Issue 12, Art no.: CD001871. doi: 10.1002/14651858. CD001871.pub3 10.1002/14651858.CD001871.pub3 22161367

[51] Campbell F, Conti G, Heckman JJ, Moon SH, Pinto R, Pungello E, Pan Y (2014). Early childhood investments substantially boost adult health. Science.

