# Supplementary material for: Higher glucose, insulin and insulin resistance (HOMA-IR) in childhood predict adverse cardiovascular risk in early adulthood: the Pune Children’s Study
Source: Diabetologia. 2015 May 5;58(7):1626–36. doi: 10.1007/s00125-015-3602-z (PMC4472941; doi:10.1007/s00125-015-3602-z)
Supplement: Supplementary file 1 — (PDF 75 kb) [file 125_2015_3602_MOESM1_ESM.pdf]

ESM Table 1: Relationship of 21-year fasting glucose, fasting insulin and HOMA-IR with 21-year CVD risk factors

	Fasting glucose	Fasting insulin	HOMA-IR
BMI	0.30 (0.16, 0.44)	0.37 (0.27, 0.47)	0.36 (0.26, 0.47)
Waist-hip ratio	0.29 (0.14, 0.43)	0.31 (0.21, 0.41)	0.30 (0.20, 0.41)
Systolic blood pressure	0.28 (0.14, 0.43)	0.12 (0.01, 0.22)	0.12 (0.01, 0.22)
Diastolic blood pressure	0.17 (0.03, 0.32)	0.21 (0.10, 0.31)	0.20 (0.10, 0.31)
Cholesterol	0.07 (-0.06, 0.21)	0.15 (0.05, 0.25)	0.15 (0.05, 0.25)
Triglycerides	0.32 (0.18, 0.46)	0.31 (0.21, 0.41)	0.32 (0.21, 0.42)
HDL cholesterol	-0.13 (-0.27, 0.01)	-0.16 (-0.27, -0.06)	-0.17 (-0.27, -0.06)
Intima media thickness	-0.02 (-0.17, 0.11)	-0.03 (-0.13, 0.07)	-0.03 (-0.14, 0.07)
Pulse wave velocity	0.17 (0.02, 0.33)	0.13 (0.02, 0.24)	0.14 (0.03, 0.25)

Values are β regression coefficients (95% CI). All dependent and independent variables in a linear regression model are Z standardized.
