# Supplementary material for: Higher glucose, insulin and insulin resistance (HOMA-IR) in childhood predict adverse cardiovascular risk in early adulthood: the Pune Children’s Study
Source: Diabetologia. 2015 May 5;58(7):1626–36. doi: 10.1007/s00125-015-3602-z (PMC4472941; doi:10.1007/s00125-015-3602-z)
Supplement: Supplementary file 2 — (PDF 84.4 kb) [file 125_2015_3602_MOESM2_ESM.pdf]

ESM Table 2: Comparison of 21-year CVD risk factors in those with and without acanthosis nigricans at 21y

	Acanthosis nigricans present (n=42) (29 boys, 13 girls)	Acanthosis nigricans absent (n=307) (156 boys, 151 girls)	p-value	p-value adjusted for gender
BMI kg/m^2	24.9 (4.5)	21.1 (3.8)	<0.001	<0.001
Waist hip ratio	0.86 (0.06)	0.82 (0.06)	0.001	0.005
Fasting glucose mmol/L	5.1 (0.3)	5.0 (0.4)	0.119	0.245
120 min glucose mmol/L †	5.7 (5.2, 7.0)	5.6 (4.8, 6.5)	0.076	0.024
Fasting insulin pmol/L †	51.7 (41.1, 87.6)	41.7 (26.6, 62.6)	0.006	<0.001
120 min insulin pmol/L †	388.2 (241.0, 786.9)	286.0 (183.7, 458.8)	0.002	0.001
HOMA IR †	1.1 (0.9, 1.8)	0.9 (0.6, 1.4)	0.005	<0.001
HOMA beta †	95.8 (83.4, 134.4)	86.9 (63.6, 109.8)	0.013	0.001
Matsuda index	4.2 (2.6, 6.1)	5.9 (3.8, 8.9)	0.001	<0.001
Insulinogenic index †	56.2 (39.2, 76.5)	53.8 (38.7, 82.0)	0.869	0.572
Systolic BP mmHg	113.3 (11.0)	108.8 (11.1)	0.015	0.138
Diastolic BP mmHg	66.5 (7.9)	64.2 (8.0)	0.094	0.144
Cholesterol mmol/L	3.7 (0.7)	3.8 (0.7)	0.474	0.393
Triglycerides mmol/L †	0.9 (0.7, 1.2)	0.7 (0.6, 1.1)	0.035	0.079
HDL cholesterol mmol/L	0.9 (0.1)	1.0 (0.2)	<0.001	0.003

Intima media thickness mm †	0.37 (0.35, 0.39)	0.36 (0.33, 0.40)	0.376	0.793
Pulse wave velocity cm/s †	1000.0 (920.0, 1073.3)	953.0 (860.0, 1036.8)	0.019	0.044

Values are mean (SD) or median (25th -75th percentiles) †. P value by t test or ANOVA or Mann-Whitney test as appropriate
