# Supplementary material for: Higher glucose, insulin and insulin resistance (HOMA-IR) in childhood predict adverse cardiovascular risk in early adulthood: the Pune Children’s Study
Source: Diabetologia. 2015 May 5;58(7):1626–36. doi: 10.1007/s00125-015-3602-z (PMC4472941; doi:10.1007/s00125-015-3602-z)
Supplement: Supplementary file 3 — (PDF 85 kb) [file 125_2015_3602_MOESM3_ESM.pdf]

ESM Table 3: Comparison of 21-year CVD risk factors in those who were in lowest quartile of HOMA-IR at both 8 and 21 years and those who were in the highest quartile at both ages

	In the lowest quartile of HOMA-IR at both 8 and 21years (n=28) (15 boys, 13 girls)	In the highest quartile of HOMA-IR at both 8 and 21years (n=31) (17 boys, 14 girls)	p-value	p-value adjusted for age, gender	p-value adjusted for age, gender, BMI, WHR
BMI kg/m^2	18.8 (3.0)	24.5 (4.4)	<0.001	<0.001	---
Waist hip ratio	0.80 (0.05)	0.86 (0.07)	0.001	<0.001	---
Fasting glucose mmol/L	4.7 (0.3)	5.4 (0.5)	<0.001	<0.001	0.002
120 min glucose mmol/L †	5.0 (4.2, 5.8)	6.0 (5.2, 7.8)	0.003	0.002	0.122
Fasting insulin pmol/L †	19.5 (14.6, 24.6)	77.7 (67.9, 100.7)	<0.001	<0.001	<0.001
120 min insulin pmol/L †	188.3 (106.9, 322.3)	488.5 (333.4, 1016.2)	<0.001	0.002	0.002
HOMA beta †	55.6 (48.9, 77.9)	117.6 (105.5, 151.0)	<0.001	<0.001	<0.001
Matsuda index†	11.1 (8.0, 16.3)	2.9 (2.0, 3.8)	<0.001	<0.001	<0.001
Insulinogenic index †	42.9 (30.4, 64.7)	70.8 (50.4, 101.8)	0.001	0.001	0.012
Systolic BP mmHg	105.8 (11.9)	113.5 (9.7)	0.009	0.003	0.570
Diastolic BP mmHg	62.4 (9.2)	67.5 (6.6)	0.018	0.016	0.689
Cholesterol mmol/L	3.6 (0.5)	4.1 (0.7)	0.002	0.003	0.009
Triglycerides mmol/L †	0.7 (0.5, 0.3)	1.2 (1.0, 1.5)	<0.001	0.001	0.091

HDL cholesterol mmol/L	1.1 (0.2)	0.9 (0.1)	0.007	0.005	0.257
Intima media thickness mm †	0.36 (0.34, 0.40)	0.36 (0.34, 0.40)	0.856	0.900	0.703
Pulse wave velocity cm/s †	926.0 (856.0, 1027.3)	975.3 (878.0, 1039.7)	0.600	0.211	0.676

Values are mean (SD) or † median (25th -75th percentiles). P value by t test or ANOVA or Mann-Whitney test as appropriate
